# Solution Blow Spun Silica Nanofibers: Influence of Polymeric Additives on the Physical Properties and Dye Adsorption Capacity

**DOI:** 10.3390/nano11113135

**Published:** 2021-11-20

**Authors:** Rosiane Maria da Costa Farias, Lucas Leite Severo, Artur P. Klamczynski, Eliton Souto de Medeiros, Lisiane Navarro de Lima Santana, Gelmires de Araújo Neves, Gregory Melvin Glenn, Romualdo Rodrigues Menezes

**Affiliations:** 1 Laboratory of Materials Technology (LTM), Department of Materials Engineering, Federal University of Campina Grande (UFCG), Av. Aprígio Veloso 882, Campina Grande 58429-900, Brazil; lucas.severo@estudante.ufcg.edu.br (L.L.S.); lisiane.navarro@ufcg.edu.br (L.N.d.L.S.); gelmires.neves@ufcg.edu.br (G.d.A.N.); 2 Western Regional Research Center, United States Department of Agriculture, Agricultural Research Service, Albany, CA 94710, USA; artur.klamczynski@usda.gov (A.P.K.); greg.glenn@usda.gov (G.M.G.); 3 Laboratory of Materials and Biosystems, Federal University of Paraiba, Cidade Universitária, João Pessoa 58051-900, Brazil; eliton@ct.ufpb.br

**Keywords:** blown spun silica nanofibers, PVP molecular weight, physical properties, dye adsorption

## Abstract

The physical properties of porous silica nanofibers are an important factor that impacts their performance in various applications. In this study, porous silica nanofibers were produced via solution blow spinning (SBS) from a silica precursor/polymer solution. Two polyvinylpyrrolidone (PVP, M_w_ = 360,000 and 1,300,000) were chosen as spinning aids in order to create different pore properties. The effect of their physical properties on the adsorption of methylene blue (MB) in an aqueous solution was explored. After forming, the nanofibers were calcined to remove the organic phase and create pores. The calcined nanofibers had a large amount of micro and mesopores without the use of additional surfactants. The molecular weight of the PVP impacted the growth of silica particles and consequently the pore size. High M_w_ PVP inhibited the growth of silica particles, resulting in a large volume of micropores. On the other hand, silica nanofibers with a high fraction of mesopores were obtained using the lower M_w_ PVP. These results demonstrate a simple method of producing blow spun silica nanofibers with defined variations of pore sizes by varying only the molecular weight of the PVP. In the adsorption process, the accessible mesopores improved the adsorption performance of large MB molecules.

## 1. Introduction

In recent years, silica nanofibers with a mesoporous structure have attracted considerable attention due to their high surface area, high pore volume and large pore size [1,2,3]. These physical properties are essential for many applications. For example, porous nanofibers comprised of different inorganic oxides proved to be effective adsorbents in the removal of toxic pollutants in wastewater presumably by facilitating the diffusion of target molecules to active sites exposed throughout the porous structure [4,5,6,7,8,9].

There are several techniques for the production of silica nanofibers, such as electrospinning (ES) [1,2] centrifugal jet spinning (CJS) [10], and solution blow spinning (SBS) [11,12]. In general, most spinning techniques used to synthesize silica nanofibers involve a sol-gel procedure that is intimately dependent on the precursor solution/sol properties and the polymeric aids used. Lately, SBS has been highlighted as a simple, affordable, versatile, high efficiency method of nanofiber production [13,14]. It is also preferred for its ability to produce nanofibers from a wide variety of polymers and solvents. SBS uses pressurized air to make fibers with a controllable and flexible morphology including highly porous nonwoven fiber mats formed by cross-layering nanofibers using a rotating collector. Nonwoven fiber mats can be useful for several applications due to the high accessibility of the pore network of the individual nanofibers and the ease of collecting and handling of fiber mats [14,15].

Typically, the mesoporous structure of the fibers can be obtained by using a template or surfactant as structure directing agent. The most common surfactants include pluronic P123 [1,7,16] and cetyltrimethylammonium bromide (CTAB) [2,17,18]. They are efficient in producing mesopore structures in several oxide nanofibers. However, their use makes the process complex and more expensive, as it is still necessary to add a spinning aid (organic polymer) to achieve a spinnable solution.

Spinning aids are essential in achieving the molecule chain entanglement required to improve the viscosity of the precursor solution and maintain a continuous jet that will reach the collector. Among the polymeric spinning aids, polyvinylpyrrolidone (PVP) is by far the most widely used for silica nanofibers production owing to its excellent chemical compatibility and stability [3,19,20]. Moreover, PVP has been used in the production of mesoporous polymer films [21] and carbon nanofibers [22] due to its amphiphilic character. Thus, due to its bifunctional properties, PVP can make the preparation and production of mesoporous silica nanofibers less complex and costly when used as a spinning aid polymer.

Studies indicate that polymer characteristics influence the physical properties of polymeric nanofibers produced by SBS [13,23]. However, there is no study on the influence of polymeric additives on the physical properties and/or dye adsorption capacity of blow spun ceramic nanofibers. Thus, the novelty of this investigation is to analyze the influence of polymers characteristics on the physical properties and dye adsorption capacity of silica fibers obtained by SBS.

To date, studies have investigated the effect of varying the amount of polymer in polymer solutions on the morphology and properties of spun polymeric fibers [24]. Molecular weight is known to affect the properties of polymer solutions as well as the properties of the nanofibers they produce. However, there is a lack of information on the effect of the molecular weight of PVP on the properties and morphology of oxide ceramic nanofibers. There are two common grades of PVP products for making silica nanofibers by electrospinning (M_w_ = 360,000 and 1,300,000). One objective of this study was to further improve the physical properties of silica nanofibers using a polymer (PVP) rather than surfactants. A further objective of this study was to compare the properties of porous silica nanofiber produced by SBS from the two common grades of PVP and without the use of surfactants. The fiber properties compared included fiber morphology, physicochemical properties, specific surface area, pore structure, and dye (methylene blue) adsorption behavior.

## 2. Experimental

### 2.1. Materials

Tetraethyl orthosilicate (TEOS, reagent 99%) and polyvinyl pyrrolidone (average M_w_ ~360,000 and M_w_ ~1,300,000) were obtained from Sigma-Aldrich (St. Louis, Mo, USA). Hydrochloric acid (HCl, Sigma-Aldrich, St. Louis, Mo, USA) and ethanol (200 proof—Pharmco-Aaper, Shelbyville, KY, USA) were used as catalyst and solvent, respectively. The cationic dye, Methylene blue (MB) (Merck Millipore, Burlington, MA, USA), was used in the adsorption experiments. The pH of dye solutions was adjusted with HCl or NaOH (1 M) (Sigma-Aldrich, St. Louis, Mo, USA). All chemicals were analytical grade and used as received.

### 2.2. Production of Nanofibers

To prepare the precursor solution, a sol containing 1 mL of TEOS, 1 mL of ethanol and 20 μL of HCl was prepared under vigorous stirring for 1 h. The mixture was then added to a solution of PVP (8 wt%) in ethanol and stirred for 1 h. The SBS apparatus consisted of a syringe pump that pumped the polymer solution through the inner nozzle of a specialized concentric nozzle. Pressure regulated air (345 kPa) was fed through the outer nozzle of the concentric nozzle. The process parameters were determined according to past experience with nanofiber formation by SBS [11,12].

The polymer solution was pumped through the inner nozzle at a rate of 110 μL min^−1^. The polymer solution was drawn into fibers by the shear effect of the pressurized air from the outer nozzle impinging on the polymer solution as it exited the inner nozzle. The jettisoned fibers passed through a heated metal tube until they reached the collector and formed a nonwoven mat. The working distance from the nozzle to the collector was 70 cm and the system temperature was controlled at approximately 60 °C. The fiber mat was kept in a lab oven (100 °C) for five days to complete condensation step. The fiber mats were calcined in air at 550 °C, using a dwell time of 3 h and heating rate of 10 °C min^−1^. The as-spun samples produced with the lower (Mw ~360,000) and the higher (Mw ~1,300,000) molecular weight PVP were denoted as SNFs/PVP-LMw and SNFs/PVP-HMw, respectively from silica nanofibers (SNF). Consequently, SNFs-LMw and SNFs-HMw were referenced to their corresponding calcined samples.

### 2.3. Characterization

The morphology of the fibers and fiber mats were analyzed by Scanning Electron Microscopy (SEM-FEG, S4700, Hitachi, Tokyo, Japan). Diameters of 100 individual fibers per sample were evaluated using ImageJ software (version 1.48, National Institutes of Health, Bethesda, MD, USA). Statistical analyses (*t* and Mann-Whitney tests) were performed at a 0.05 level of significance. Thermal gravimetric analysis (Pyris 1 model, Perkin-Elmer, Norwalk, CT, USA) was employed to evaluate the weight loss of the samples tested under a nitrogen atmosphere (20 mL min^−1^) at a heating rate of 10 °C min^−1^ from 30 to 700 °C. The FTIR spectra (Thermo Fisher Scientific, Waltham, MA, USA, KBr pellet (2/100 mg)) were recorded in the spectral range of 4000 to 400 cm^−1^, with 32 scanning and 4 cm^−1^ resolutions. X-ray diffraction (X’Pert Philips diffractometer, Malvern Panalytical, Malvern, UK) spectra were obtained using a CuKα anode (λ = 0.15406 nm) with a scanning step of 0.02° in the range of 10–60°. Elemental analysis (CHNS Elemental Analyzer, model 2400, Perkin Elmer, Akron, OH, USA) was used to determine CHN content. The physical properties for the silica nanofibers were determined from N_2_ adsorption/desorption experiments performed at 77 K (ASAP 2020, Micromeritics, Norcross, GA, USA). Brunauer-Emmett-Teller (BET) surface area was calculated from adsorption data and the Barrett-Joyner-Halenda (BJH) model was used to calculate the pore volume. The micropore volume and micropore surface area were obtained by the *t*-plot method [25] and Zeta potential values (nano-ZS, Malvern Instruments, Malvern, UK) were measured and recorded. Surface charge was analyzed by varying the pH from 3 to 10.

### 2.4. Adsorption Evaluation

Aqueous dye stock solutions (1.0 g L^−1^) were prepared for use in adsorption tests. Adsorption tests were performed by placing 5 mg of the fiber sample in flasks containing MB solution (10 mL) with initial concentration of 100 mg L^−1^. The pH of the solutions was controlled by 1 M HCl and 1 M NaOH in the range of 4, 5–10. The isotherm of adsorption of MB was performed by a series of solution at initial concentrations varying from 10 to 500 mg L^−1^ at pH 10, the tests were repeated three times. The solutions were stirred on an orbital shaker (MaxQ 4000, Thermo Scientific, Malvern, UK) at 250 rpm for 24 h while keeping the temperature constant at 298 K. After each experiment, the solution (1 mL) was taken and centrifuged (5000 rpm, 5 min) to determine the residual concentration of the dye. The concentration of the MB was measured by UV-Vis spectrophotometer at *λ_max_* 664 nm. The effect of pH on the adsorption of dyes was investigated at 298 K. Similar experiments were carried out at 308 and 318 K to determine the thermodynamic data. The effect of contact time and kinetic studies were carried out using a solution concentration of 100 mg L^−1^ for MB (pH 10). For this purpose, dye solution was added with the adsorbent at 298 K. The dye concentration was measured at various time intervals (15–480 min). The adsorption capacity was calculated using Equation (1) and the percentage removal efficiency was calculated using Equation (2). Equation variables were defined where *Q_e_* is the maximum capacity (mg g^−1^), *C*_0_ and *C_f_* are initial and final dye concentrations, respectively, *V* is the volume of the dye solution (L) and *m* is the amount of the adsorbent (mg).

(1) $$Q_{e}=\left(C_{0}-C_{f}/m\right)V$$

(2) $$\%\;Removal=(C_{0}-C_{f}/C_{0})100$$

#### 2.4.1. Adsorption Isotherm Studies

The adsorption isotherm parameters obtained from the linear fitting of the Langmuir and Freundlich models were evaluated in order to determine the correlation between the amount of MB adsorbed onto the studied adsorbents. The Langmuir isotherm assumes uniform active sites and monolayer adsorption of molecules on the adsorbent surface. Mathematically, this is expressed using the linearized Langmuir Equation (3).

(3) $$C_{e}/Q_{e}=\left(C_{e}/Q_{e}\right)+\left(1/Q_{m}K_{L}\right)$$

where *Q_m_* represents the maximum adsorption (mg g^−1^), *Q_e_* is the amount adsorbed at equilibrium (mg g^−1^), *C_e_* is the dye solution concentration at equilibrium (mg L^−1^), *K_L_* is the Langmuir constant (L mg^−1^) which is equivalent to the equilibrium constant of chemical reactions.

The Freundlich isotherm is applied in adsorption studies under heterogeneous surface energy and multilayer adsorption and can be expressed according to the linear Equation (4).

(4) $$lnQ_{e}=lnK_{f}+\left(1/n\right)lnC_{e}$$

where *C_e_* is the concentration of dye at equilibrium (mg L^−1^), *K_f_* [(mg g^−1^) (L mg^−1^) 1/*n*] and 1/*n* represent the Freundlich constants, *K_f_* represents an approximate value of the adsorption capacity. The intensity of the adsorption process is represented as 1/*n* and is related to the heterogeneity of the surface where values between 0 and 1 are obtained and where values closer to 0 indicate a more heterogeneous surface.

#### 2.4.2. Thermodynamic Studies

Thermodynamic parameters such as Gibbs free energy change (Δ*G*⁰), entropy change (Δ*S*⁰) and enthalpy change (Δ*H*^0^) were calculated from Equations (5)–(7). Δ*G*⁰ (J/mol) was estimation from Equation (5) by using *K* (dimensionless) as thermodynamic equilibrium constant. In this case, *K* was calculated as dimensionless by multiplying *K_L_* (L mg^−1^) as equilibrium constant (obtained from Langmuir model) by *M_adsorbate_* (molecular weight of adsorbate, mg mol^−1^) and by 55.5 (water concentration, mol L^−1^) (See Equation (6)) [26]. The slope and intercept obtained from the linear fitting (ln*K* versus 1/*T*) of Van’t Hoff Equation (7) was used to calculate Δ*S*⁰ and Δ*H*⁰. In both Equations (5) and (7) *T* is the temperature in Kelvin and *R* is the ideal gas constant (8.314 J mol^−1^ K^−1^).

(5) $$\Delta G^{0}=-RTlnK$$

(6) $$K=K_{L}\times M_{adsorbate}\times 10^{3}\times 55.5)$$

(7) $$lnK=-\Delta H^{0}/RT+\Delta S^{0}/R$$

#### 2.4.3. Kinetic Studies

Equations (8)–(10) were used to investigate the mechanism of MB adsorption by fitting the experimental data through the kinetics models (i.e., pseudo-first-order, pseudo-second-order, and intraparticle diffusion, respectively).

(8) $$\ln\left(Q_{e}-Q\right)=lnQ_{e}-k_{1}t$$

(9) $$t/Q_{t}=1/k_{2}Q_{e}^{2}+1/Q_{e}t$$

(10) $$Q_{t}=k_{dif}^{1/2\;}+C$$

where *Q_e_* is the amount of dye adsorbed (mg g^−1^) at equilibrium, *Q_t_* is the amount of dye adsorbed at a determined time, *t* is the contact time (min), *k*_1_ (min^−1^), *k*_2_ (g mg^−1^ min^−1^) and *K_dif_* (mg g^−1^ min^−1/2^) are the rate constant for the pseudo-first-order, pseudo-second-order and intraparticle diffusion, respectively. The *C* (mg g^−1^) parameter represents is the amount of dye adsorbed and is determined from the intercept of the linearized *Q_t_* versus *C* in Equation (10).

#### 2.4.4. Statistical Parameters

Each model was validated for the adsorption of dyes onto the nanofibers through the applicable fit verified using correlation coefficients (*R*^2^), as well as the root mean square error (*RMSE*) and the standard deviation *SD* given in Equations (11) and (12), respectively.

(11) $$RMSE=\sqrt{\sum {\left(Q_{e,exp}-Q_{e,cal}\right)}^{2}/n}$$

(12) $$SD=\sqrt{\sum {\left(Q_{e,exp}-Q_{e,cal}\right)}^{2}/n-1}$$

where *Q_e,cal_* and *Q_e,exp_* refer to the calculated and experimental data, and *n* is the total number of data points. The value of *R*^2^ closest of the unit and the lower values of *RMSE* and *SD* indicate the best fit of the model.

## 3. Results

**Figure 1:**
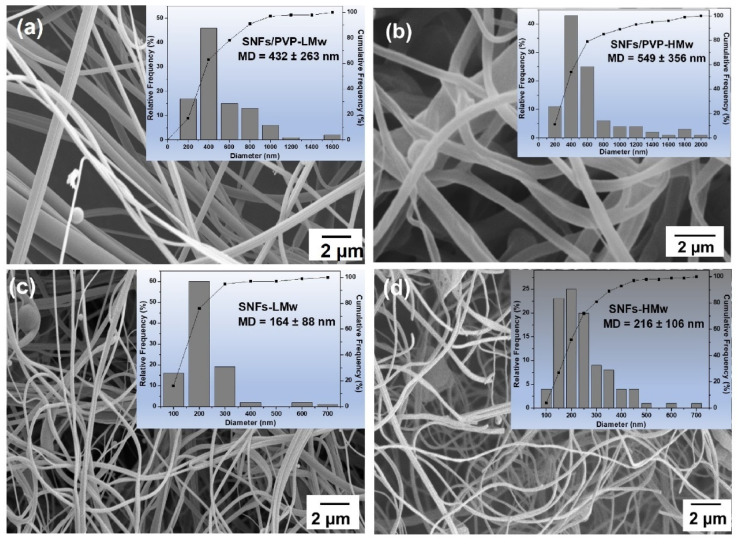
SEM images and distribution of the diameter (inset) of: (**a**) SNFs/PVP-LMw, (**b**) SNFs/PVP-HMw, and (**c**) SNFs-LMw and (**d**) SNFs-HMw.

Silica nanofibers (SNFs) were successfully prepared via the SBS technique with the aid of PVP as a porogenic agent and spinning aid. Figure 1a–d shows the morphologies of the as-spun and calcined nanofibers at different PVP molecular weight (360,000 and 1,300,000). SEM images show a structure comprised of randomly oriented nanofibers with a cylindrical and thin, elongated shape. The mean diameter of SNFs/PVP-LMw and SNFs/PVP-HMw was 432 ± 263 nm (Figure 1a inset) and 549 ± 356 nm (Figure 1c inset), respectively. Figure 1b,d show that the fibrous structure of the samples were well preserved after the calcination process. The mean diameters of the fibers were slightly affected by the differences the molecular weight of the PVP. Thus, the mean diameter of SNFs/PVP-LMw is lower than SNFs/PVP-HMw (*t* test, *p* < 0.001). There is a significant shrinkage in fiber diameter (*t* test, *p* < 0.001) due thermal degradation/removal of the organic polymer component and a consolidation/densification process for both samples. The fiber diameters were reduced to 164 ± 88 nm for SNFs-LMw (Figure 1b inset) and to 216 ± 106 nm for SNFs-HMw (Figure 1d inset). The mean diameter was lower in SNFs-LMw relative to the SNF-HMw fibers (*t* test*, p* < 0.001). However, differences in the mean fiber diameter between SNFs/PVP-LMw and SNFs/PVP-HMw were not significantly different (Mann-Whitney test, *p* = 0.0918), while differences in the range of fiber diameter s of calcinated samples are different (Mann-Whitney test, *p* < 0.001).

**Figure 2:**
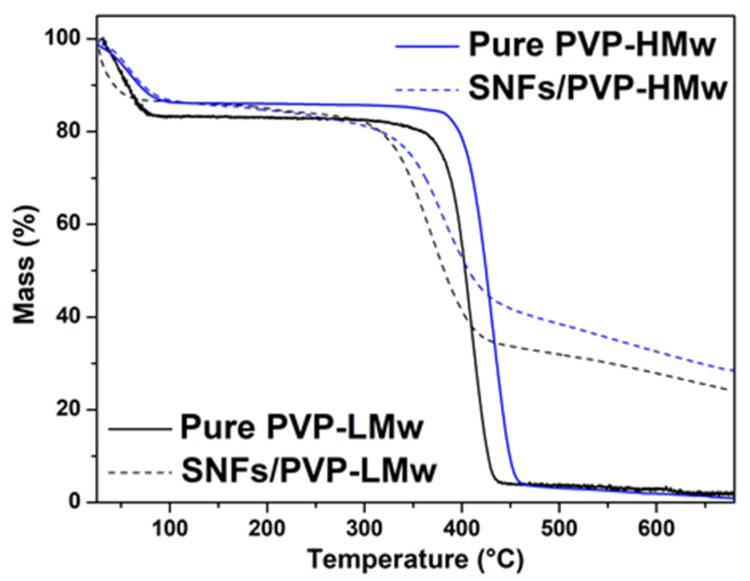
TGA curves of SNFs/PVP-LMw and SNFs/PVP-HMw.

Figure 2 shows the thermal behaviors of pure PVP-LMw (M_w_ = 360,000), PVP-HMw (M_w_ = 1,300,000), and as-spun nanofibers (SNFs/PVP-LMw and SNFs/PVP-HMw). For pure PVP, the TGA curves exhibit two stages of mass loss. An initial stage with the maximal mass loss occurred approximately at 100 °C, which can be attributed to the evaporation of physiosorbed water. In this stage, mass loss is greater for PVP-LMw (~17%) than for PVP-HMw (~14%). The lower physiosorbed moisture in PVP-LMw can be attributed to its lower molecular weight and concomitant higher surface area on which to absorb water. Moreover, the thermal degradation of PVP chains (observed in the second stage) was lower for PVP-LMw (from 310 °C to 440 °C) than PVP-HMw (from 340 °C to 465 °C). This is also a function of molecular weight and chain entanglement since the greater entanglement of the PVP-HMw molecular chains can make its decomposition more difficult. In this stage of thermal degradation, C–H, C–C, C = O, and C–N bonds are broken and degraded into carbon oxide and nitrogen oxide compounds [27].

The size of the polymer chains also has a significant importance in the TGA curves of as-spun nanofibers. The curves revealed two stages of mass loss with the first occurring near 100 °C and was interpreted as a loss of physiosorbed water and residual free ethanol from the condensation of hydrolyzed TEOS to silica [27]. For SNFs/PVP-LMw, the evaporation phase of free liquid started at a lower temperature than that for SNFs/PVP-HMw. This was presumably due to the lower degree of chain entanglement which allowed the solvent molecules a less tortuous path for evaporation.

**Scheme 1:**
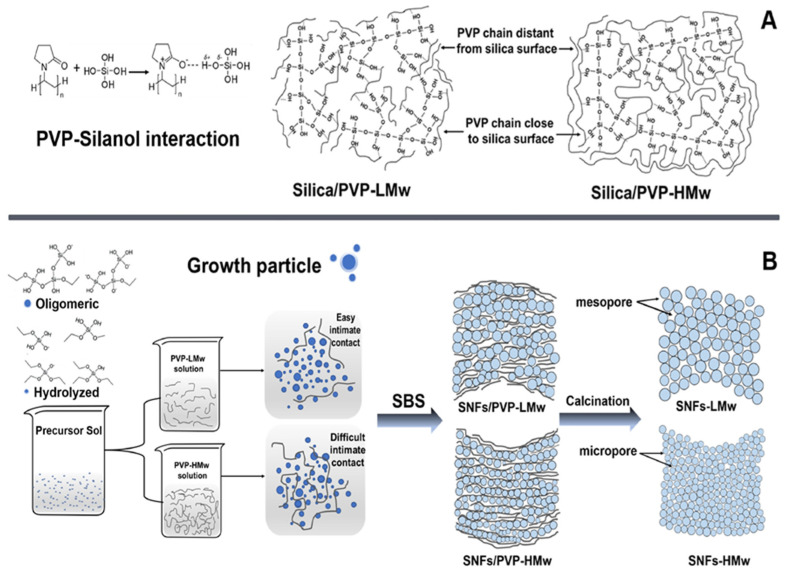
(**A**) Interaction between silanol groups and PVP unit and chain, and (**B**) schematic illustration of PVP chain and silica particle in precursor solution, as-spun fiber and pores size after calcination process.

The second stage of mass loss occurred near 320 °C where the PVP in the hybrid fibers exhibited lower thermal stability than pure PVP. According to Bogatyrev et al. [28], the interaction of PVP and silica occurs only through the hydrogen bonds between the carbonyl (C = O) group in PVP and the silanol (SiO-H) group on the silica surface (Scheme 1A). This interaction can weaken the pyrrolidone ring bonds and their degradation can occur at lower temperatures. A greater mass loss during the second stage occurred between 300 °C and 435 °C, where carbon dioxide and pyrrolidone rings were the main components released. Over this temperature range, a continual loss of mass was observed for both SNFs/PVPs, which was believed to involve the thermal degradation of PVP chains closest to the surface of the silica phase [28,29,30] and the release of water in the silica framework by the condensation of silanols groups [31]. Mass loss was found to be slightly greater for SNFs/PVP-LMw (70%) than SNFs/PVP-HMw (64%) up to 550 °C.

This difference suggests that high M_w_ chains favor a higher packing and association with the silica network compared to low M_w_ PVP where more intimate carbonyl-silanol interactions may occur (Scheme 1A). It may be that the depolymerization of PVP at high temperatures may be slowed by the number of polymer units bound with silanol groups on the silica surface [28]. Hence, the residual mass is composed of the silica phase with small amounts of carbon. In contrast, low molecular weight PVP chains may have a greater intimate contact with ≡ SiOH groups to reach the oligomeric forms. Then, the condensation rate of the silanol groups was more pronounced, which released a greater amount of water and increased the size of the silica particles (Scheme 1B).

Table 1 shows the carbon (C), hydrogen (H), and nitrogen (N) content of the samples which verified the presence of polymer residues in the silica nanofibers after calcination. The results confirm that both samples have similar N content. However, the amount of C was three times greater for SNFs-HMw than for SNFs-LMw.

**Figure 3:**
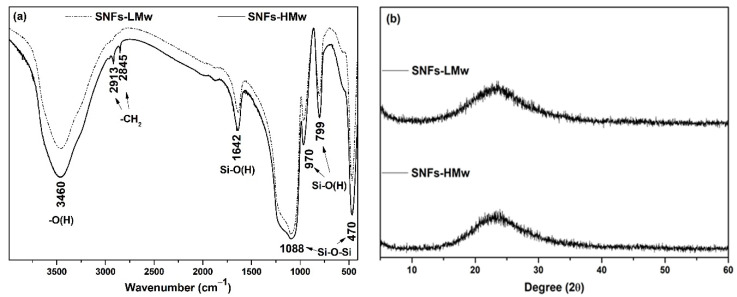
(**a**) FT-IR spectra and (**b**) XRD pattern of SNFs-LMw and SNFs-HMw.

FT-IR analysis of the SNFs-LMw and SNFs-HMw are shown in Figure 3a. The spectra of both samples show characteristic IR bands of amorphous silica. The broad band between 3100 and 3700 cm^−1^, centered at 3460 cm^−1^ is attributed to O-H stretching frequency related to physiosorbed water and silanol groups on silica surface [32]. The symmetric and antisymmetric Si-O-Si (siloxane) stretching are observed at 1080 and 470 cm^−1^, respectively. Stretching vibration at 970 cm^−1^ and bending frequency at 799 cm^−1^ correspond to the surface silanol groups Si-O(H) [33,34,35,36]. However, the spectra for SNFs-HMw clearly showed peaks at 2913 and 2845 cm^−1^ which was attributed to CH_2_ absorption of PVP which confirmed that the existence of residual carbon content was more significant in this sample. The XRD analysis (Figure 3b) indicated the presence of amorphous silica for both calcinated samples, with only a small broad band in the 2-theta range of 15–30° [19,20].

**Figure 4:**
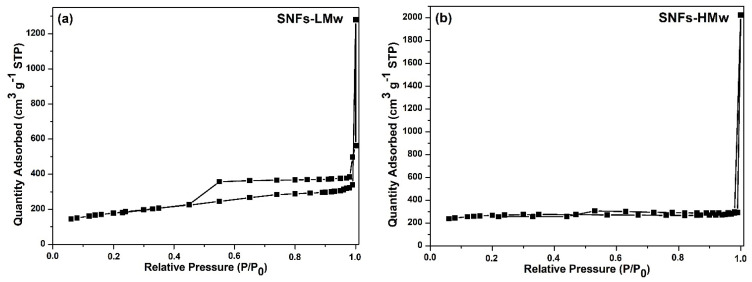
N_2_ adsorption–desorption isotherm of: (**a**) SNFs-LMw and (**b**) SNFs-HMw.

N_2_ adsorption-desorption isotherms for SNFs-LMw shown in Figure 4a are identified as type IV, indicating a mesoporous material according to IUPAC classification. In the low relative pressure region (*p*/*p*_0_ < ~0.1) the uptake of nitrogen can be observed, which indicates the presence of micropores. Mesoporous structures are also observed when the relative pressure (*p*/*p*_0_) is more than ~0.5 which is associated with capillary condensation. This is where the nitrogen uptake increases and starts the typical H4 hysteresis loop [37] and indicates the presence of narrow slit pores [38,39]. On the other hand, plotted isotherms of SNFs-HMw can be classified as composites of type I + IV isotherms with a discreet hysteresis loop type H4 (See Figure 4b). Isotherms type I are typical of microporous materials [40], which have high surface areas.

The physical parameters of the samples are summarized in Table 2. BET analysis indicated the largest surface area (921.7 m^2^ g^−^^1^) for SNFs-HMw while in SNFs-LMw samples had a lower surface area in comparison (635.6 m^2^ g^−1^), which was indicative of mostly micropores. This result was confirmed by the *t*-plot results in which the micropore volume and micropore area were much high for SNFs-HMw than for SNFs-LMw. In contrast, BJH desorption cumulative volume of pore width between 1.7 nm and 300 nm indicates a high pore volume (0.74 cm^3^ g^−1^) and desorption average pore width of 3.67 nm for SNFs-LMw. For SNFs-HMw those values are 0.12 cm^3^ g^−1^ and 1.99 nm, respectively (Table 2).

The results of physical characterization indicate that both samples contained micro/mesopores. However, SNFs-LMw was predominantly comprised of mesoporous pores and SNFs-HMw was predominantly microporous in nature. This can be explained by the different sizes of the polymer layers, which impacted the PVP-silanol and silanol-silanol interactions. As mentioned above, the lower M_w_ PVP favored a greater intimate contact between the silanol groups. This contributed to the formation of nuclei that favored the growth of silica particles by the hydrolysis/condensation step, resulting in large particles. Therefore, large particles favored the formation of larger pores (Scheme 1B).

**Figure 5:**
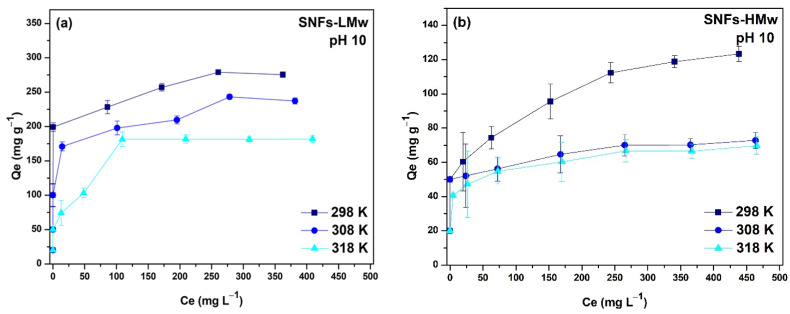
Effect of initial MB concentration and temperature: (**a**) SNFs-LMw and (**b**) SNFs-HMw. Error bars represent standard deviations of triplicate tests.

Based on isotherm data (the data represent the mean of three experiments and the error bars represent the standard deviations of triplicate tests (Figure 5a,b), the adsorption yield onto both adsorbents increased as initial MB concentration increased. This can be attributed to the high number of dye molecules colliding with the adsorbent, which provided a driving force that overcame the mass transfer resistance between them. On the other hand, the adsorption capacity decreased as the temperature increased, indicating an exothermic process. The influence of the physical properties of SNFs-LMw and SNFs-HMw on the adsorption capacity of MB was clearly demonstrated. Despite the higher specific surface area of SNFs-HMw, SNFs-LMw had higher adsorption capacity. As shown in Figure 5a,b, the maximum adsorption capacity of SNFs-LMw and SNFs-HMw at 298 K is 278.86 mg g^−1^ and 123.29 mg g^−1^, respectively. These differences can be attributed to the properties of the pores. The predominant mesoporous structure of SNFs-LMw may have facilitated the transport of the MB molecule into the porous fiber structure where more sites for adsorption could be accessed. In contrast, the molecular dimensions of MB molecule (1.70 nm × 0.76 nm × 0.33 nm) [41] is close to the average pore diameter of SNFs-HMw (1.99 nm), which may have made MB access to binding sites more difficult (resulting in less uptake).

**Figure 6:**
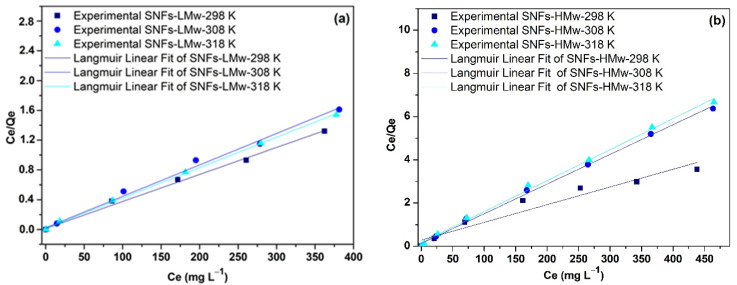
The Langmuir isotherm and linear fit for MB adsorbed by: (**a**) SNFs-LMw and (**b**) SNFs-HMw at 298 K, 308 K, and 318 K.

Table 3 shows the results obtained from the adsorption parameters derived according to the linear forms of the two models (Equations (3) and (4)). The statistical parameters were also explored to identify the fit quality of each model. The best fit model was chosen based on the highest correlation coefficients (*R*^2^) and the lowest *SD* and *RMSE* values. Comparing the results, the adsorption of MB on both adsorbents was best fit by the Langmuir isotherm. Figure 6a,b show the experimental and Langmuir linear fit (best fit model) obtained when Ce/Qe was plotted against Ce at 298 K, 308 K and 318 K for SNFs-LMw and SNFs-HMw, respectively. *Q_max(calc)_* and *K_L_* were evaluated from the slope and intercept. The *Q_max(calc)_* of SNFs-LMw and SNFs-HMw is 275.5 mg g^−1^ and 121.9 mg g^−1^, which are very close to the experimental values (*Qe*). These results means that a monolayer coating of MB molecules was formed on the energetically homogeneous surface of the nanofibers. Therefore, the differences in the physical properties of the samples did not change the adsorption behavior of the dyes.

**Figure 7:**
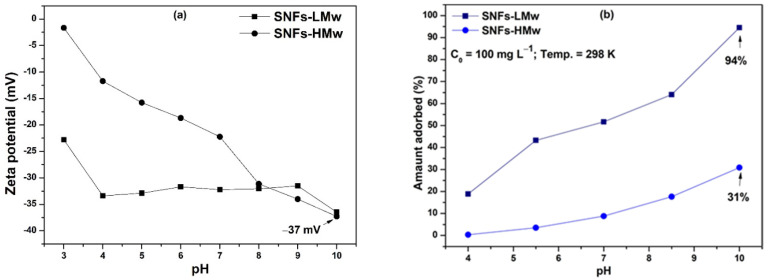
Comparative results of SNFs-LMw and SNFs-HMw adsorbents of (**a**) zeta potential and (**b**) pH effect of MB adsorption.

Zeta potential values of the adsorbents were determined varying the pH from 3 to 10 in order to verify the surface charge. Moreover, silanolate groups (SiO**^−^**) were expected to be the prevalent silica form in a large pH range due to the low isoelectric point of the silica surface (IEP ≈ 2–3) [41]. As shown in Figure 7a, for both adsorbents the surface is negatively charged for the range of all pH conditions tested. SNFs-HMw becomes more negative as the pH increases, and no significant change is observed for SNFs-LMw between pH 4 and 10. Thus, SNFs-LMw exhibited higher density of SiO^−^ groups even at low pH conditions. Although SNFs-HMw has a significant carbon content that can modify the surface charge, there was no charge difference between HMw and LMw fibers at pH greater than 8. The high content of hydroxyl groups on the surface of the silica fibers surface develops negative charges in aqueous solutions, notably at high OH**^−^** concentrations. This means that the degree of deprotonation of hydroxyl groups increases at a high pH [33].

Under high pH conditions, the silanol groups are deprotonated and become negatively charged. Hence, ≡Si-O^−^ can attract and bind the positively charged MB^+^ molecules. However, SNFs-HMw shows a low adsorption capacity in comparison to SNFs-LMw under all pH conditions (Figure 7b). Despite data showing that SNFs-HMw has a higher BET surface area and a zeta potential at pH 10 similar to the SNFs-LMw, the adsorption capacity of MB onto SNFs-LMw is three times higher than that of SNFs-HMw at this pH. This means that the amount of silanolate groups and total surface area are not the main factors that determine the adsorption capacity. Since SNFs-LMw have a much higher mesopore volume than SNFs-HMw, it is possible that mesopores are an important factor in governing the adsorption of MB dye. Indeed, MB binding may be strongly influenced by the combination of the physical characteristics of the SNFs and the high density of the SiOH groups.

**Figure 8:**
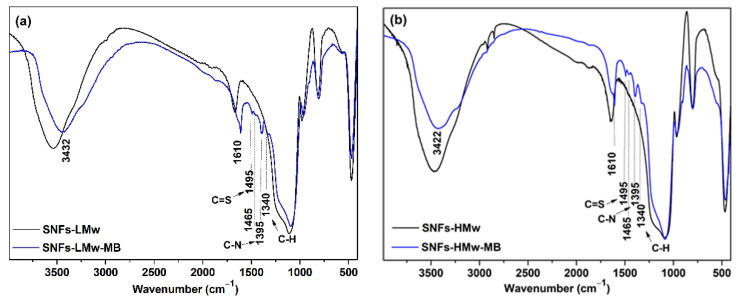
FTIR spectra before and after MB adsorption for (**a**) SNFs-LMw and (**b**) SNFs-HMw.

Figure 8 shows FTIR analysis of SNFs-LMw (Figure 8a) and SNFs-HMw (Figure 8b) after adsorption of MB (blue line) in comparison with the adsorbents before removal (black line). For SNFs-LMw-MB and SNFs-HMw-MB, the peak at 1642 cm^−1^ corresponds to Si-O(H) shifted to 1610 cm^−1^. In addition, the -O(H) vibration peak centered at 3460 cm^−1^ also shifted and corresponds to 3432 cm^−1^ and 3422 cm^−1^ for SNFs-LMw and SNFs-HMw, respectively. These results may be attributed to the interaction between MB molecules and the adsorbents, through hydrogen bonds [42]. Furthermore, the new small peaks attributed to the stretching vibration of -C-N, -C-H and C = S in the MB molecule are shown at 1334 cm^−1^, 1395 cm^−1^, and 1492 cm^−1^, respectively [43,44].

**Figure 9:**
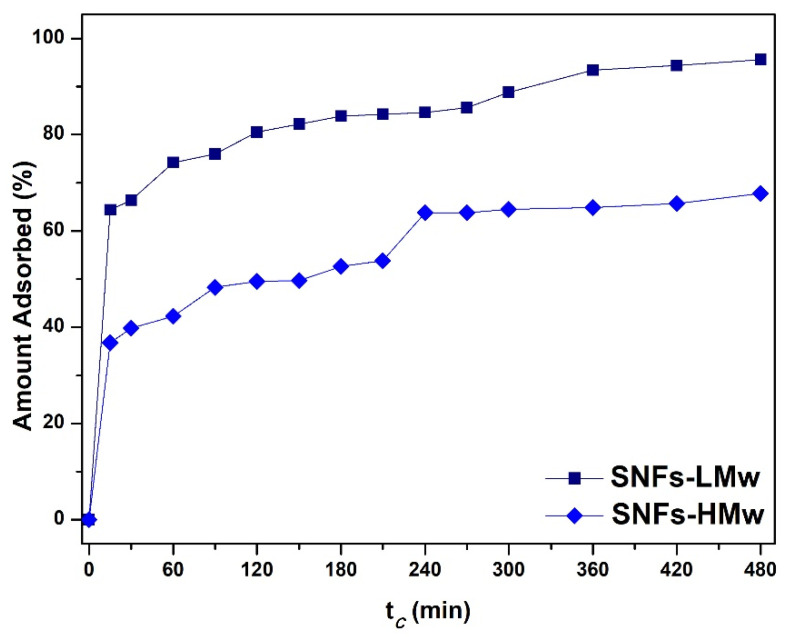
Effect of contact time on MB adsorption onto SNFs-LMw and SNFS-HMw.

The adsorption of MB on both SNFs was evaluated at different time intervals in the range of 15 to 480 min (Figure 9). As can be seen, approximately 65% of the MB adsorbed occurred in the first 15 min for SNFs-LMw. The rapid uptake could be due to an initial adsorption occurring on the external surface and on the larger mesopores of the adsorbent. For SNFs-HMw which have only a small fraction of mesopores, only 37% of MB was adsorbed at the first 15 min. Consequently, adsorption was relatively slow as the dye molecules diffused into the smaller pores. The adsorption equilibrium was achieved after 360 min for SNFs-LMw, which reached ~96% of MB removal. Adsorption equilibrium for SNFs-HMw occurred after 240 min with only ~65% of MB removal.

The kinetic parameters were determined by adjusting the kinetic data of the MB adsorption in the models of pseudo-first-order, pseudo-second-order and intra-particle diffusion. The results are summarized in Table 4. Intra-particle-diffusion occurred during the adsorption process due to porosity and high surface area. However, the kinetic constant of *K_dif_*, and the relatively low *R*^2^ value confirmed that Intra-particle-diffusion is not the sole rate-limiting step for the adsorption process [18]. For pseudo-first-order and pseudo-second-order models, the values of the experimental adsorption capacity (*Q_e(exp)_*) were closer to the calculated adsorption capacity (*Q_e(cal)_*) by pseudo-second-order models for both adsorbents. The results confirm second-order adsorption kinetics best fit the adsorption behavior. This is in agreement with the high *R*^2^ values and the lower values of SD and RMSE and indicate that the chemisorption process is the main rate controlling step for MB dye adsorption for both SNFs.

Table 5 shows energy (Δ*G*⁰), entropy (Δ*S*⁰) and enthalpy change (Δ*H*⁰) estimated for both MB adsorption onto SNFs-LMw and SNFs-HMw at 298 K, 308 K and 318 K. Results show the influence of physical properties of the fibers on the thermodynamic parameters. Negative values and positive values of Δ*H*⁰ indicating that the adsorption process is exothermic and endothermic for SNFs-LMw and for SNFs-HMw, respectively. This is in agreement with the results in adsorption isotherms for SNFs-LMw, in which the adsorption capacity decreased with increasing temperature. For SNFs-HMw, the adsorption capacity with increasing temperature from 308 K to 318 K may not be considerable. The positive values of ΔS⁰ reflect the increase in randomness at the solid/liquid interface during adsorption for both SNFs. The more negative values of ΔG⁰ for SNFs-LMw indicate an adsorption more spontaneous and energetically favorable than that for SNFs-HMw.

Table 6 shows the comparison with the results of previous studies on MB adsorption capacities available in the literature [8,16,45,46,47,48,49]. Most of them are mesoporous materials with high surface area. This is a demonstration of the efficiency of mesoporous materials for application as dye adsorbents. Therefore, the SNFs-LMw adsorption capacity is higher than those observed in literature. This indicates that the improved adsorption capacity is attributed to the superior physical properties of the sample.

## 4. Conclusions

Mesoporous silica nanofibers were obtained by the SBS technique. Nanofibers with different specific surface area and pores features were obtained by using PVP of different M_w_. The results revealed that SNFs contained mesopore and micropores. SNFs produced using PVP with lower Mw were rich in mesopores. while SNFs produced using PVP with higher M_w_ were rich in micropores. The PVP samples differed in the average chain length which likely governed the pore diameter. Although micropores allow the connection between mesopores. the high content of micropores negatively impacted MB removal. Adsorption tests showed the same behavior and mechanism. including electrostatic interaction of MB onto both SNFs-LMw and SNFs-HMw. However. the physical properties of SNFs-LMw made it a more promising adsorbent for MB dye molecules.

## Figures and Tables

**Table 1:** Elemental analysis.

Sample	C[%]	H[%]	N[%]
SNFs-LMw	0.65	1.71	0.54
SNFs-HMw	1.86	1.29	0.56

**Table 2:** Physical parameters of SNFs-LMw and SNFs-HMw.

Sample	BET [m^2^ g^−1^]	BJH Desorption ^(a)^ [cm^3^ g^−1^]	Pore Diameter ^(b)^ [nm]	Micropore Volume ^(c)^ [cm^3^ g^−1^]	Micropore Area ^(d)^ [m^2^ g^−1^]	External Surface Area ^(e)^ [m^2^ g^−1^]
SNFs-LMw	635.6	0.747	3.67	0.05	118.9	516.7
SNFs-HMw	921.7	0.122	1.99	0.23	510.3	411.3

^(**a**)^ (BJH Desorption cumulative volume of pores between 1.7000 nm and 300.0000 nm width); ^(**b**)^ (Desorption average pore width); ^(**c**)^ (*t*-Plot micropore volume); ^(**d**)^ (*t*-Plot Micropore Area); ^(**e**)^ (*t*-Plot External Surface Area).

**Table 3:** Langmuir and Freundlich parameters of SNFs-LMw and SNFs-HMw for MB derivate from isotherms adsorption.

Sample	Temperature [K]	Langmuir					
		*Qe* [mg g^−1^]	*Q_max_* _(cal)_ [mg g^−1^]	*K_L_* [L mg^−1^]	*R* ^2^	*SD* [%]	*RMSE*
SNFs-LMw	298 308 318	278.8 250.0 243.2	275.5 247.6 238.1	0.29 0.29 0.19	0.996 0.999 0.994	0.41 0.22 0.32	1.17 0.30 5.10
SNFs-HMw	298 308 318	123.3 72.9 69.9	121.9 72.8 69.7	0.05 0.11 0.09	0.987 0.997 0.997	0.18 0.01 0.03	0.49 0.01 0.03
		Freundlich					
		1/*n*	*K_F_* [mg g^−1^] [L mg^−1^]		*R* ^2^	*SD* [%]	*RMSE*
SNFs-LMw	298 308 318	0.14 0.10 0.14	122.4 126.7 141.2		0.883 0.867 0.897	39.12 30.82 25.50	78.2 24.7 20.4
SNFs-HMw	298 308 318	0.13 0.17 0.31	31.6 25.1 16.5		0.808 0.812 0.852	15.34 9.56 8.90	37.4 7.9 7.6

**Table 4:** Kinetic modeling results of MB adsorption onto SNFs-LMw and SNFs-HMw.

	Pseudo-First-Order Model					
Sample	*Q_e(exp)_* [mg g^−1^]	*Q_e(calc)_* [mg g^−1^]	*K_1_* [10^−3^ min^−1^]	*R* ^2^	*SD*	*RMSE*
SNFs-LMw	191.20	80.40	7.12	0.881	30.73	29.61
SNFs-HMw	135.67	80.22	7.34	0.913	15.38	14.82
	**Pseudo-Second-Order Model**					
		***Q_e(calc)_* [mg g^−1^]**	***K_2_* [10^−4^ g mg^−1^ min^−1^]**	***R* ^2^**	***SD***	***RMSE***
SNFs-LMw	191.20	181.82	4.42	0.998	2.51	2.42
SNFs-HMw	135.6	140.80	2.09	0.983	1.39	1.34
	**Intra-Particle Diffusion**					
		***C* [mg g^−1^]**	***K_dif_* [mg g^−1^ min^−1/2^]**	***R* ^2^**	***SD***	***RMSE***
SNFs-LMw	191.20	118.64	11.83	0.971	20.12	19.39
SNFs-HMw	135.6	58.65	14.04	0.938	21.34	20.56

**Table 5:** Thermodynamic parameters of MB adsorption on SNFs-LMw and SNFs-HMw.

	SNFs-LMw			SNFs-HMw		
T (K)	298	308	318	298	308	318
ln*K*	15.44	15.45	15.03	13.70	14.45	14.26
Δ*G*⁰ (kJ mol^−1^)	−38.25	−39.56	−39.74	−33.94	−35.98	−37.70
Δ*H*⁰ (kJ mol^−1^)	−15.49			21.16		
Δ*S*⁰ (J mol^−1^ K^−1^)	76.90			186.30		

**Table 6:** Comparison of the MB adsorption capacity of different adsorbents.

Adsorbent	BET [m^2^ g^−1^]	BJH Desorption [cm^3^ g^−1^]	Pore Diameter [nm]	Q_e_ (mg g^−1^)	References
Mesoporous carbon nanofibers	392.3	-	3–5	58.992	[8]
SBA15 *Silica gel Mesoporous carbon* MCSG60	780 440 650	-	7 6 8	~20 ~20 ~200	[16]
Porous carbon nanofibers	885.551	0.967	3.928	123.64	[45]
Graphene	295.56	-	3.49	153.85	[46]
N-doped mesoporous carbon-	166.9	-	3.2 and 6.0	163	[47]
meso/macro-porous silica microspheres coated with polydopamine MSM@PDA	612.3	0.91	6 and 100	83.30	[48]
γ-Fe_2_O_3_/SiO_2_	74.35	-	-	116.09	[49]
*SNFs-LMw*	635.6	0.747	3.67	278.8	This work
*SNFs-HMw*	921.7	0.122	1.99	123.3	This work

## Data Availability

Not applicable.

## References

[1] Noh W., Kim T.H., Lee K.-W., Lee T.S. (2020). Selective adsorption of sodium dodecylbenzenesulfonate from a Cs ion mixture by electrospun mesoporous silica nanofibers. Chemosphere.

[2] Kim S., Park H., Choi H. (2019). Maneuvering the ordered mesoporosity of electrospun silica nanofibers for water harvesting. Microporous Mesoporous Mater..

[3] Diagboya P.N., Dikio E.D. (2018). Silica-based mesoporous materials. emerging designer adsorbents for aqueous pollutants removal and water treatment. Microporous Mesoporous Mater..

[4] Aguado J., Arsuaga J.M., Arencibia A., Lindo M., Gascón V. (2009). Aqueous heavy metals removal by adsorption on amine-functionalized mesoporous silica. J. Hazard. Mater..

[5] Vu D., Li Z., Zhang H., Wang W., Wang Z., Xu X., Dong B., Wang C. (2012). Adsorption of Cu(II) from aqueous solution by anatase mesoporous TiO_2_ nanofibers prepared via electrospinning. J. Colloid Interface Sci..

[6] Gan Y., Tian N., Tian X., Ma L., Wang W., Yang C., Zhou Z., Wang Y. (2015). Adsorption behavior of methylene blue on amine-functionalized ordered mesoporous alumina. J. Porous Mater..

[7] Shen J., Li Z., Wu Y.-N., Zhang B., Li F. (2015). Dendrimer-based preparation of mesoporous alumina nanofibers by electrospinning and their application in dye adsorption. Chem. Eng. J..

[8] Li S., Jia Z., Li Z., Li Y., Zhu R. (2016). Synthesis and characterization of mesoporous carbon nanofibers and its adsorption for dye in wastewater. Adv. Powder Technol..

[9] Yu Z., Xu C., Yuan K., Gan X., Zhou H., Wang X., Zhu L., Zhang G., Xu D. (2018). Template-free synthesis of MgO mesoporous nanofibers with superior adsorption for fluoride and Congo red. Ceram. Int..

[10] Ren L., Ozisik R., Kotha S.P. (2014). Rapid and efficient fabrication of multilevel structured silica micro-/nanofibers by centrifugal jet spinning. J. Colloid Interface Sci..

[11] Farias R.M.D.C., Severo L.L., da Costa D.L., de Medeiros E.S., Glenn G.M., Santata L.N.D.L., Neves G.D.A., Kiminami R.H.G.A., Menezes R.R. (2018). Solution blow spun spinel ferrite and highly porous silica nanofibers. Ceram. Int..

[12] Farias R.M.D.C., Mota M.F., Severo L.L., de Medeiros E.S., Klamczynski A.P., Avena-Bustillos R.D.J., Santana L.N.D.L., Neves G.A., Glenn G.M., Menezes R. (2020). Green Synthesis of Porous N-Carbon/Silica Nanofibers by Solution Blow Spinning and Evaluation of Their Efficiency in Dye Adsorption. J. Mater. Res. Technol..

[13] Medeiros E.S., Glenn G.M., Klamczynski A.P., Orts W.J., Mattoso L.H.C. (2009). Solution blow spinning: A new method to produce micro- and nanofibers from polymer solutions. J. Appl. Polym. Sci..

[14] Daristotle J.L., Behrens A.M., Sandler A., Kofinas P. (2016). Review of the Fundamental Principles and Applications of Solution Blow Spinning. ACS Appl. Mater. Interfaces.

[15] Lim C.T. (2017). Nanofiber technology: Current status and emerging developments. Prog. Polym. Sci..

[16] Gómez J., Galán J., Rodriguez A., Walker G. (2014). Dye adsorption onto mesoporous materials: pH influence. kinetics and equilibrium in buffered and saline media. J. Environ. Manag..

[17] Zhu G.-T., Chen X., He X.-M., Wang H., Zhang Z., Feng Y.-Q. (2015). Electrospun Highly Ordered Mesoporous Silica–Carbon Composite Nanofibers for Rapid Extraction and Prefractionation of Endogenous Peptides. Chem. Eur. J..

[18] Cherifi Z., Boukoussa B., Mokhtar A., Hachemaoui M., Zeggai F.Z., Zaoui A., Bachari K., Meghabar R. (2020). Preparation of new nanocomposite poly(GDMA)/mesoporous silica and its adsorption behavior towards cationic dye. React. Funct. Polym..

[19] Calisir M., Kilic A. (2020). A comparative study on SiO2 Nanofiber Production via Two Novel Non-Electrospinning Methods: Centrifugal Spinning vs Solution Blowing. Mater. Lett..

[20] Shahhosseininia M., Bazgir S., Joupari M.D. (2018). Fabrication and investigation of silica nanofibers via electrospinning. Mater. Sci. Eng. C.

[21] Udayabhaskar R., Mangalaraja R., Manikandan D., Arjunan V., Karthikeyan B. (2012). Room temperature synthesis and optical studies on Ag and Au mixed nanocomposite polyvinylpyrrolidone polymer films. Spectrochim. Acta A Mol. Biomol. Spectrosc..

[22] Li X., Zhao Y., Bai Y., Zhao X., Wang R., Huang Y., Liang Q., Huang Z. (2017). A Non-Woven Network of Porous Nitrogen-doping Carbon Nanofibers as a Binder-free Electrode for Supercapacitors. Electrochim. Acta.

[23] Oliveira J.E., Moraes E.A., Costa R.G.F., Afonso A.S., Mattoso L.H.C., Orts W.J., Medeiros E.S. (2011). Nano and submicrometric fibers of poly(D.L-Lactide) obtained by solution blow spinning: Process and solution variables. J. Appl. Polym. Sci..

[24] Rotta M., Zadorosny L., Carvalho C., Malmonge J., Malmonge L.F. (2016). YBCO ceramic nanofibers obtained by the new technique of solution blow spinning. Ceram. Int..

[25] Harkins W.D., Jura G. (1944). Surfaces of solids: XIII. A vapor adsorption method for the determination of the area of a solid without the assumption of a molecular area. and the areas occupied by nitrogen and other molecules on the surface of a solid. J. Am. Chem. Soc..

[26] Anastopoulos I., Kyzas G.Z. (2016). Are the thermodynamic parameters correctly estimated in liquid-phase adsorption phenomena?. J. Mol. Liq..

[27] Shi W., Lu W., Jiang L. (2009). The fabrication of photosensitive self-assembly Au nanoparticles embedded in silica nanofibers by electrospinning. J. Colloid Interface Sci..

[28] Bogatyrev V.M., Borisenko N.V., Pokrovskii V.A. (2001). Thermal Degradation of Polyvinylpyrrolidone on the Surface of Pyrogenic Silica. Russ. J. Appl. Chem..

[29] Bianco G., Soldi M., Pinheiro E., Pires A., Gehlen M., Soldi V. (2003). Thermal stability of poly(N-vinyl-2-pyrrolidone-co-methacrylic acid) copolymers in inert atmosphere. Polym. Degrad. Stab..

[30] Jablonski A.E., Lang A.J., Vyazovkin S. (2008). Isoconversional kinetics of degradation of polyvinylpyrrolidone used as a matrix for ammonium nitrate stabilization. Thermochim. Acta.

[31] Zhao Y., Wang H., Lu X., Li X., Yang Y., Wang C. (2008). Fabrication of refining mesoporous silica nanofibers via electrospinning. Mater. Lett..

[32] Kim M., Kang S.-H., Manuel J., Zhao X., Cho K.K., Ahn J.H. (2015). Investigation into the role of silica in lithium polysulfide adsorption for lithium sulfur battery. Mater. Res. Bull..

[33] Temel F., Turkyilmaz M., Küçükçongar S. (2020). Removal of methylene blue from aqueous solutions by silica gel supported calix [4] arene cage: Investigation of adsorption properties. Eur. Polym. J..

[34] Hu X., Wang X., Liu J., Zhang S., Jiang C., He X. (2012). Fabrication of mesoporous dendritic silica nanofibers by using dendritic polyaniline templates. Mater. Chem. Phys..

[35] Wu S., Li F., Wang H., Fu L., Zhang B., Li G. (2010). Effects of poly (vinyl alcohol) (PVA) content on preparation of novel thiol-functionalized mesoporous PVA/SiO_2_ composite nanofiber membranes and their application for adsorption of heavy metal ions from aqueous solution. Polymer.

[36] Yue X., Feng S., Li S., Jing Y., Shao C. (2012). Bromopropyl functionalized silica nanofibers for effective removal of trace level dieldrin from water. Colloids Surf. A Physicochem. Eng. Asp..

[37] Yang H., Elma M., Wang D., Motuzas J., da Costa J.C.D. (2017). Interlayer-free hybrid carbon-silica membranes for processing brackish to brine salt solutions by pervaporation. J. Membr. Sci..

[38] Lowell S., Shields J.E., Thomas M.A., Thommes M. (2004). Characterization of Porous Solids and Powders: Surface Area. Pore Size and Density.

[39] Alothman Z.A. (2012). A Review: Fundamental Aspects of Silicate Mesoporous Materials. Materials.

[40] Ghimire P., Gunathilake C., Wickramaratne N.P., Jaroniec M. (2017). Tetraethyl orthosilicate-assisted synthesis of nitrogen-containing porous carbon spheres. Carbon.

[41] Araghi S.H., Entezari M.H. (2015). Amino-functionalized silica magnetite nanoparticles for the simultaneous removal of pollutants from aqueous. Appl. Surf. Sci..

[42] Ji Y., Xu F., Wei W., Gao H., Zhang K., Zhang G., Xu Y., Zhang P. (2021). Efficient and fast adsorption of methylene blue dye onto a nanosheet MFI zeolite. J. Solid State Chem..

[43] Ovchinnikov O.V., Evtukhova A.V., Kondratenko T., Smirnov M., Khokhlov V.Y., Erina O.V. (2016). Manifestation of intermolecular interactions in FTIR spectra of methylene blue molecules. Vib. Spectrosc..

[44] Alver E., Metin A., Brouers F. (2020). Methylene blue adsorption on magnetic alginate/rice husk biocomposit. Int. J. Biol. Macromol..

[45] Wang J., Cai C., Zhang Z., Li C., Liu R. (2020). Electrospun metal-organic frameworks with polyacrylonitrile as precursors to hierarchical porous carbon and composite nanofibers for adsorption and catalysis. Chemosphere.

[46] Liu T., Li Y., Du Q., Sun J., Jiao Y., Yang G., Wang Z., Xia Y., Zhang W., Wang K. (2012). Adsorption of methylene blue from aqueous solution by graphene. Colloids Surf. B Biointerfaces.

[47] Liang T., Wang F., Liang L., Liu M., Sun J. (2016). Magnetically separable nitrogen-doped mesoporous carbon with high adsorption capacity. J. Mat. Sci..

[48] Germi T.A., Nematollahzadeh A. (2016). Bimodal porous silica microspheres decorated with polydopamine nano-particles for the adsorption of methylene blue in fixed-bed columns. J. Colloid Interface Sci..

[49] Chen D., Zeng Z., Zeng Y., Zhang F., Wang M. (2016). Removal of methylene blue and mechanism on magnetic γ-Fe_2_O_3_/SiO_2_ nanocomposite from aqueous solution. Water Resour. Ind..

